# How Does Teachers’ Psychological Capital Influence Workplace Well-Being? A Moderated Mediation Model of Ego-Resiliency and Work-Meaning Cognition

**DOI:** 10.3390/ijerph192214730

**Published:** 2022-11-09

**Authors:** Binghai Sun, Hongteng Guo, Luyun Xu, Fujun Ding

**Affiliations:** 1 Research Center of Tin Ka Ping Moral Education, Zhejiang Normal University, Jinhua 321004, China; 2 College of Teacher Education, Zhejiang Normal University, 688 Yingbin Road, Jinhua 321004, China; 3 College of Education and Human Development, Zhejiang Normal University, Jinhua 321004, China; 4 Key Laboratory of Intelligent Education Technology and Application of Zhejiang Province, Zhejiang Normal University, Jinhua 321004, China

**Keywords:** teachers’ psychological capital, ego-resiliency, work-meaning cognition, workplace well-being

## Abstract

Previous studies found that teachers’ psychological capital positively affects their workplace well-being. However, the underlying internal mechanism behind this relationship remains ambiguous. The current study aimed to investigate the effects of ego-resiliency and work-meaning cognition on this relationship among Chinese teachers. The questionnaire, including the psychology capital scale (PCS), workplace well-being subscale (WWBS), Psychological Empowerment Scale (PESS), and Ego-Resiliency Scale (ERS), was used to collect data points from 1388 primary and secondary school teachers. The results reveal that: (1) teachers’ psychological capital positively predicts workplace well-being; (2) work-meaning cognition mediates the relationship between teachers’ psychological capital and workplace well-being; (3) the influence of work-meaning cognition on the relationship between teachers’ psychological capital and workplace well-being is moderated by ego-resiliency. These findings explore the factors that affect well-being and point to potential ways to enhance teachers’ workplace well-being.

## 1. Introduction

### 1.1. The Importance of Workplace Well-Being for Teachers

As times advance, the importance of knowledge and talents becomes more and more prominent, and the status and role of education have also increased [1,2]. In education, teachers are the organizers and leaders of education and play a bridge role in the continuation and development of human society [3,4,5]. However, surveys show that most teachers have some degree of psychological problems, often feeling the effects of a heavy workload and psychological pressure, which seriously influences the quality of teaching [6,7]. Some researchers propose that workplace well-being is a pivotal index to measure the mental state of teachers [8]. Workplace well-being refers to the employee’s feelings about their work; the positive emotions they feel in work experience are more than negative [9,10]. It is a comprehensive evaluation of the degree of subjective pleasure of the individual in the work experience, encompassing social, emotional, physical, and economic factors [11]. In China and even around the world, the mental health problems of teachers have always attracted the attention of researchers, especially the well-being of teachers [12].

It is crucial to focus on teachers’ mental and physical health, measured by workplace well-being. Previous studies show that teachers’ workplace well-being is closely related to the improvement of teachers’ self-development and quality of life, and it is one of the determinants of teachers’ professional growth, success, motivation, and professional activities [13,14]. In addition, a growing body of evidence demonstrates that teachers’ workplace well-being also has considerable influence on classroom teaching quality, students’ academic performance, and their emotional development [15,16,17,18]. The sense of workplace well-being in a teacher’s profession is the key content of their occupational life and an important index to measure the quality of their working life [19,20]. Therefore, attention should be paid to teachers’ workplace well-being. Not only does it contribute to improving the quality of teachers’ working life, but also it enhances the quality of education.

### 1.2. Teachers’ Psychological Capital and Workplace Well-Being

Some empirical research has shown that psychological capital can predict workplace well-being [21,22,23]. Psychological capital is a general term for various positive mental states including self-efficacy (confidence), hope, optimism, tenacity, emotional intelligence and so on, which play a catalytic role in the process of personal growth and performance improvement [24,25]. It could also reflect employees’ job involvement and retention intention [26,27,28]. Xu et al. explored the impact of different types of capital on workplace well-being [21]. The results show that psychological capital could also have a key effect on achieving a high level of well-being, even without human capital and social capital. Motivation theory holds that needs generate motivation, and individual motivation can be activated only when the intrinsic potential of a person develops to an extent that could meet certain needs [29]. Therefore, individuals with higher psychological capital will hold higher expectations for the job and think that they are competent enough to perform the job, so as to maintain long-term enthusiasm and motivation. Driven by this positive state and persistent motivation, their inner potential will be further stimulated, thus, showing higher happiness and job performance at work [30,31]. The present study focused on exploring how teachers’ psychological capital could affect workplace well-being, which might support development of effective interventions.

### 1.3. Teachers’ Psychological Capital, Work-Meaning Cognition, and Workplace Well-Being

The relationship between teachers’ psychological capital and workplace well-being may be mediated by work-meaning cognition. Work-meaning cognition is defined as employees feeling about whether their work is significant and has positive valence [32]. Recent studies on workplace well-being indicate that work-meaning cognition can predict workplace well-being [33,34]. Moreover, the studies also found that it is linked to work burnout, work engagement, retirement anxiety, and other mental health problems [34,35]. Lavy and Naama found that when teachers possess a higher level of work-meaning cognition, they tend to be more active in caring about students, and build a good relationship between teachers and students, to obtain a higher level of well-being at work.

At the same time, some theories of work-meaning hold that low psychological capital is a risk factor leading to a lack of work-meaning cognition. Sutrisno et al. simultaneously built an atheoretical model including work-meaning cognition and psychological capital. They found a significant and positive correlation between psychological capital and work-meaning cognition; the individual with little psychological capital possessed less cognition of work-meaning. These theories and studies show that work-meaning cognition might be an essential mediator, affecting the relationship between teachers’ psychological capital and workplace well-being. Hence, we hypothesized that the relationship between teachers’ psychological capital and workplace well-being might be mediated by work-meaning cognition (H2).

### 1.4. Teachers’ Psychological Capital, Ego-Resiliency, Work-Meaning Cognition, and Workplace Well-Being

The relationship between teachers’ psychological capital and work-meaning cognition may be moderated by ego-resiliency. Ego-resiliency refers to the ability of individuals to develop and adapt well even when faced with tremendous pressure [36]. Ego-resiliency is vital to developing social competence and key to forming and maintaining mental health [37,38]. Previous studies also found low ego-resiliency related to low empathy, low workplace well-being, depression, and anxiety [39,40,41]. In addition, work-meaning cognition belongs to the sense of life meaning. Steger pointed out in his research that the sense of life meaning has a positive predictive effect on resilience [42]. The higher the sense of meaning, the higher the psychological resilience is after a traumatic experience. Moreover, there is a positive correlation between psychology capital and ego-resiliency, which is also proven in previous research [43,44]. Specifically, higher ego-resiliency could amplify the effect of psychology capital on work engagement, safety compliance, coping strategies, and mental health [45,46,47]. Li et al. explained the mechanisms of employee workplace well-being from a self-determination perspective.

The self-determination theory holds that when individuals’ basic problems are solved, they have more opportunities to establish better goals, which could foster intrinsic engagement and employee workplace well-being [48,49]. In this way, ego-resiliency as a self-regulating ability, which could help them recover quickly from daily difficulties, might also boost the beneficial effects of work-meaning cognition. Therefore, we presumed that a high level of ego-resiliency might enhance the relationship between teachers’ psychological capital and workplace well-being via work-meaning cognition (H3).

### 1.5. Purpose of the Present Study

**Figure 1:**
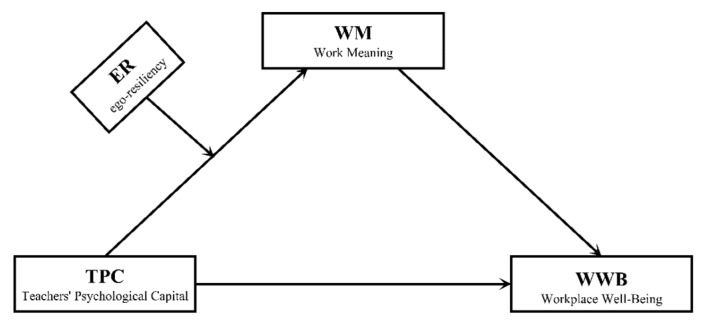
*Hypothetical Model of four main variables*. Note: TPC, teachers’ psychological capital; ER, ego-resiliency; WM, work-meaning cognition; WWB, workplace well-being.

Some empirical research shows that teachers’ psychological capital is related to workplace well-being [21,22,23]. However, the internal mechanisms between teachers’ psychological capital and workplace well-being are still unknown. Hence, the first aim of the current study was to investigate the relationships among teachers’ psychological capital, work-meaning cognition, and workplace well-being. The second aim was to examine a moderated mediation model where ego-resiliency moderates the relationship between teachers’ psychological capital and workplace well-being via work-meaning cognition. The moderated mediation model has unique advantages compared with the simple model, which could comprehensively consider various factors and test the underlying processes of how predictor factors affect outcome factors. Therefore, we built a moderated mediation model to investigate the internal mechanism among these factors. The theoretical model is detailed in Figure 1.

Based on these existing theories and empirical evidence, we hypothesized: (1) teachers’ psychological capital would be positively related to workplace well-being. (2) Work-meaning cognition would mediate teachers’ psychological capital and workplace well-being. (3) Ego-resiliency would play a moderator role between teachers’ psychological capital and workplace well-being via work-meaning cognition. In particular, a high level of ego-resiliency would enhance the mediating influence of work-meaning cognition on the effect of teachers’ psychological capital on workplace well-being.

## 2. Method

### 2.1. Sample

We conducted stratified sampling with school type and teaching grade as stratification variables, but only in Zhejiang province. In total, 1388 data points from primary and secondary school teachers were obtained by Credamo, a Chinese data collection platform (URL: www.credamo.com). These questionnaires we used were an assignment that teachers must complete when they participated in vocational training, so our questionnaire response rate reached 100%. Before they answer, we would ensure the anonymity and confidentiality of their responses. This study was approved by the University Committee on Human Research Protection at Zhejiang Normal University and was carried out in accordance with the approved guidelines (Protocol code: 20210069, approved 1 April 2021). After collecting and sorting, the average age of teachers was 39.40, and the standard deviation was 8.84. In addition, female teachers accounted for 72.10%, and male teachers accounted for 27.90%. Table 1 shows more detailed demographic statistics about the participants.

### 2.2. Instrument

The study used psychology capital scale to measure the psychological capital of teachers [50]. This scale was specific for primary and secondary school teachers. Nineteen items, which were divided into four factors, were on the scale. The four factors were: confidence (4 items, e.g., “I believe I am competent in teaching.”), hope (4 items, e.g., “I am full of energy to complete the teaching goals set by myself at present.”), optimism (5 items, e.g., “I feel optimistic and happy almost every day.”), and resiliency (6 items, e.g., “No matter how hard the teacher’s work is, I will stick to it.”). Each item’s answer ranged from 1 (*strongly disagree*) to 6 (*strongly agree*). This scale had good reliability and validity in China [51]. The Cronbach’s *α* was 0.92 in the current study.

Teachers’ workplace well-being was assessed by the subscale of the employee well-being, which Zheng developed with a Chinese sample [10]. Six items of the workplace well-being subscale (WWBS) were chosen to survey in the current study. The participants’ responses ranged from 1 (strongly disagree) to 5 (strongly agree). The highest scores of the respondents in the scale represent the highest workplace well-being among the teachers. This scale was adopted in this study because it was developed in a Chinese context and has been proven with higher validity and reliability [52]. The Cronbach’s α coefficient of this scale in the current study was 0.95.

The work-meaning cognition was one of the dimensions of psychological empowerment and measured with the corresponding dimension, including 3 items, in the Psychological Empowerment Scale [53]. A sample item includes: “The work I do is very meaningful to me”. A 5-point Likert scale was used to respond to the items (1 = *strongly disagree*, 5 = *strongly agree*). Higher scores meant perceiving more meaning in the workplace. The scale was tested to have good reliability and validity in China [54]. The Cronbach’s *α* was 0.94 in our study.

The Ego-Resiliency Scale (ERS) was utilized to assess the trait of ego-resilience [55]. Fourteen items were in the scale and scored on a 5-point Likert-type response format (1 = *does not apply at all,* 5 = *applies very strongly*). A sample item includes: “I am generous with my friends”. Higher scores signified that participants had a greater trait of self-resilience. Block et al. reported that the scale’s α coefficient was 0.76, which indicated good reliability and validity [55,56,57]. This scale’s Cronbach’s *α* was 0.92 in our research.

### 2.3. Data Analysis

In this study, SPSS21.0 was adapted for data analysis and processing. Firstly, the common method bias was analyzed to exclude the effects from self-reporting. Then, correlation analysis was used to explore the relationships among these main variables. On this basis, we performed the PROCESS macro of SPSS (model 4) to examine the mediating effects of work-meaning cognition on this relationship. Finally, PROCESS macro of SPSS (model 7) was used to test the moderated mediation effects of ego-resiliency on the relationship between teachers’ psychological capital and workplace well-being via work-meaning cognition.

## 3. Results

### 3.1. Common Method Bias

All questionnaires used were self-reported by the study subjects, so there may be common method bias. Herman’s single factor analysis was performed with the eigenvalues set as 1, and it extracted six factors, explaining 64.37% of the total variation. The first factor explained 42.76% of variance, which was no more than half of the total variance explanation. Therefore, this study has no serious common method bias problem [58]. In addition, confirmatory factor analysis (CFA) was performed using Mplus8.3, and the fitting index was as follows: χ2/df = 50.095 > 3, RMSEA = 0.108 > 0.1, CFI = 0.685 < 0.9, TLI = 0. 669 < 0.9, and SRMR = 0.082 > 0.05. The fitting index of this model does not meet the standard of good fit, indicating that there is no serious common method bias in the data of this study.

### 3.2. Correlation Statistics

Correlation analyses were performed using the main variables in this study, including teachers’ psychological capital, ego-resiliency, work-meaning cognition, and workplace well-being. We found that teachers’ psychological capital is positively associated with ego-resiliency, work-meaning cognition, and workplace well-being. Also, ego-resiliency is positively associated with work-meaning cognition, and workplace well-being. In addition, the analyses present a positive correlation between work-meaning cognition and workplace well-being. The mean and standard deviation of each variable and the correlation between variables are shown in Table 2. All bivariate correlations are statistically significant (*p* < 0.01).

### 3.3. Model of Work-Meaning Cognition as a Mediator

The present study performed the PROCESS macro of SPSS (Model 4) to test the mediating effect of work-meaning cognition on teachers’ psychological capital to workplace well-being. The results show that teachers’ psychological capital is positively related to work-meaning cognition (*β* = 0.43, *p* < 0.001) and workplace well-being (*β* = 0.64, *p* < 0.001), which supports Hypothesis 1. Meanwhile, work-meaning cognition is also positively related to workplace well-being (*β* = 0.51, *p* < 0.001). The standardized indirect effect of teachers’ psychological capital on workplace well-being via work-meaning cognition is significant, indirect effect = 0.33, SE = 0.02, 95% CI = [0.28, 0.38], and the indirect effect accounts for 43.42% of the total effect. The results show that work-meaning cognition moderates the relationship between teachers’ psychological capital and workplace well-being through its positive relationship with teachers’ psychological capital, which supports Hypothesis 2. More details about the analyses can be seen in Table 3.

### 3.4. Testing the Moderated Mediation Model

To test the mediating path of “teachers’ psychological capital (X) → work-meaning cognition (M) → workplace well-being (Y)”, moderated mediation analysis was carried out to determine whether the variable of access to the ego-resiliency (W) played a moderating role in the mediating path. According to the method proposed by Hayes, SPSS plug-in PROCESS model 7 was selected for testing [59]. The results show (Table 4) that teachers’ psychological capital positively predicts work-meaning cognition (*β* = 0.65, *p* < 0.001) and positively predicts workplace well-being (*β* = 0.43, *p* < 0.001). At the same time, the interaction term of teachers’ psychological capital and ego-resiliency also significantly predicts work-meaning cognition (*β* = −0.12, *p* < 0.001, 95%CI = [−0.19, −0.06]). It indicates that the care-going experience has a moderating effect on the first half of this mediation path.

**Figure 2:**
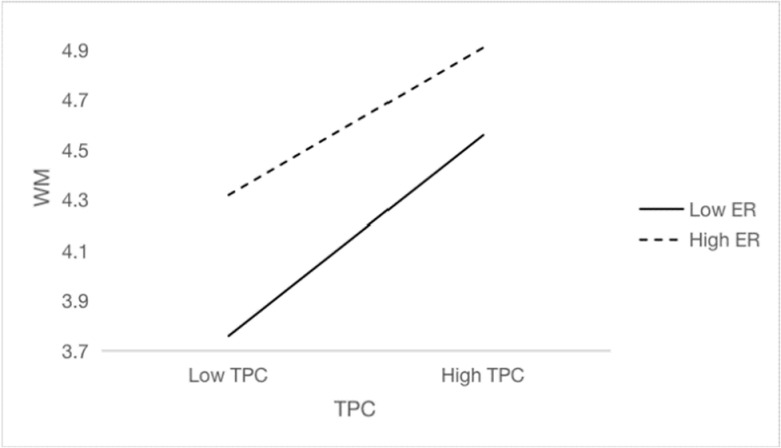
The moderating effect of ego-resiliency on teachers’ psychology capital and work-meaning cognition.

**Figure 3:**
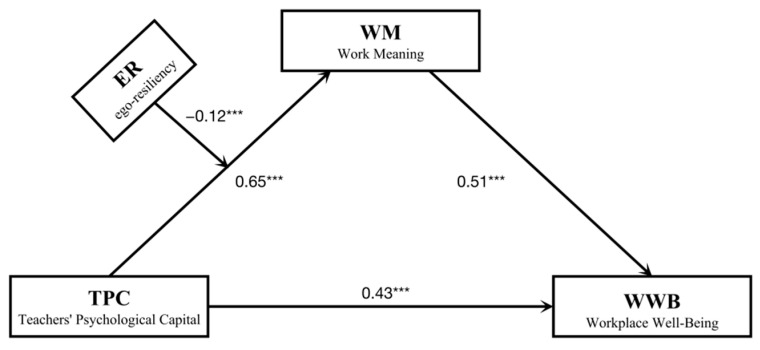
The moderated mediation model of four main variables. *** *p* < 0.001.

Further simple slope tests show that ego-resiliency has a moderating effect on the relationship between teachers’ psychological capital and work-meaning cognition (*β* = 1.00, *p* < 0.001, 95%CI = [0.69, 1.32]). We compared the effects of teachers’ psychological capital on work-meaning cognition under different levels of self-resilience and plotted simple effect analysis chart (see Figure 2). When the level of ego-resiliency is low, teachers’ psychological capital has a significant positive predictive effect on work-meaning cognition, *β*_simple_ = 0.53, *t* = 18.60, *p* < 0.001, 95%CI = [0.47, 0.58]. When the level of ego-resiliency is higher, the predictive of teachers’ psychological capital is smaller, *β*_simple_ = 0.38, *t* = 11.40, *p* < 0.001, 95%CI = [0.31, 0.44]. These results suggest that ego-resiliency plays a moderating role in the first half of the mediating path of “teachers’ psychological capital (X) → work-meaning cognition (M) → workplace well-being (Y)”. Based on these results, we construct a moderated mediation model (see Figure 3), which supports Hypothesis 3.

## 4. Discussion

Previous studies support the idea that there exists a connection between teacher’s psychological capital and workplace well-being where teachers’ psychological capital positively predicts workplace well-being. However, its underlying mechanisms still need to be explored in depth. In this study, we aimed to examine whether teachers’ psychological capital positively predicts workplace well-being through work-meaning cognition and whether the level of ego-resiliency moderates this process. The results reflect that the predictive effect of high teachers’ psychological capital on workplace well-being is partially explained by work-meaning cognition and enhanced by ego-resiliency. Teachers’ psychological capital could positively predict work-meaning cognition for respondents with low ego-resiliency. When the level of ego-resiliency is high, the positive effect of teachers’ psychological capital becomes smaller. As a result, we constructed a moderated mediation model based on these findings.

### 4.1. Relationship between Teachers’ Psychological Capital and Workplace Well-Being

We found a significant total effect of teachers’ psychological capital on workplace well-being, which supported the findings of previous studies [21,22,23]. Luthans’s psychological capital theory proposes that psychological capital consists of four positive factors (self-efficacy, optimism, hope, and resilience), which are critical aspects of employee and organizational success [60]. Individuals with high psychological capital might feel less burnout and be more tolerant of colleagues’ uncivilized behaviors [61,62]. Further, Laschinger and Fida found that psychological capital could reduce the feelings of job stress [63]. Employees who experience fewer negative emotions tend to gain more occupational satisfaction and workplace mental health. According to psychological capital theory, psychological capital is a type of positive psychological state. With high levels of psychological capital, individuals are more likely to focus on the positive aspects of their surroundings against challenges from their lives [64]. Based on psychological capital theory, psychological capital could help employees deal with various life pressures and dedicate their energies to work [65]. In this way, they will improve their work engagement and feel more workplace well-being [66]. Therefore, if individuals possess high levels of psychological capital, they will perform more positively and be more hopeful, making psychological capital an indicator of teachers’ career prosperity [67].

### 4.2. The Mediating Role of Work-Meaning Cognition

A mediation model was constructed to test the indirect effect of work-meaning cognition on the relationship between teachers’ psychological capital and workplace well-being. As hypothesized, work-meaning cognition plays a mediating role in the influence of teachers’ psychological capital on workplace well-being. Work-meaning cognition refers to employees’ subjective understanding of the work’s meaning [68]. Seligman constructed the PERMA model, including five dimensions (positive emotions, engagement, relationships, meaning, and accomplishment), which are conceptualized as essential to well-being [69]. Based on PERMA model, Goh et al. examined the effect mechanism of positive relationships and work-meaning cognition [70]. Their results show that assisting employees in discovering their work meaning is a more economical and feasible strategy of cultivating positive emotions in the workplace.

Moreover, Sirgy proposed that workplace well-being involves job satisfaction and work-related affects [71]. The work-related affects means emotions experienced at work, which could be accumulated during the exploration of work-meaning. In this research, work-meaning cognition largely explains the predictive effect of teachers’ psychological capital on workplace well-being, which is consistent with the results of prior studies [33,34]. These results again support the psychological capital theory that teachers with more psychological capital find more meaning in their work [60]. Thus, they acquire more workplace well-being compared to the low psychological capital individuals.

### 4.3. The Moderating Role of Ego-Resiliency

More importantly, we observe that ego-resiliency might enhance the relationship between teachers’ psychological capital and workplace well-being via work-meaning cognition. Therefore, Hypothesis 3 of the present study is finally confirmed. Based on conservation of resources theory (COR), employees’ workplace well-being could be improved by preventing resource consumption and supporting staffs’ access to resources [72]. Besides stress, an external factor that strongly negatively impacts workplace well-being is employee resilience, which was added as an internal factor to the theoretical framework of COR by Iqbal and Piwowar-Sulej [73]. Their study found the predictive role of employee resilience for employees’ well-being. However, there is little in the literature concerned the integrated relationship between teachers’ psychological capital, ego-resiliency, work-meaning recognition, and workplace well-being. Therefore, we successfully constructed a moderation model that comprehensively considered these four variables in this study, confirming the COR theory and enriching the literature of the relevant fields [73].

In brief, ego-resiliency could moderate the predictive effect of teachers’ psychological capital on workplace well-being through work-meaning recognition. Specifically, teachers with relatively high ego-resiliency would be more positive and recover quickly when they face stressful situations, thus, mitigating the negative impact of low psychological capital and social support. In addition, teachers with better ego-resiliency possess more opportunities to explore work meaning, which, in turn, increases the level of workplace well-being.

### 4.4. Limitations and Future Research

There are several limitations to this study. First, all of our questionnaires asked participants to self-report, which may influence the study’s validity because of the social expectation. Future studies should employ more objective measures of teachers’ psychological capital and workplace well-being, such as using implicit association, applying other-report, and collecting objective behavioral data. Second, the study was designed as a cross-sectional survey, which could hardly make inferences about causality only by explaining the correlation between variables. Hence, future research could perform further interaction analysis on the data, or adapt longitudinal designs to examine the casual relationships among the main variables. Third, female teachers accounted for 72.10% of our sample, far higher than the males, which created a gender imbalance in our study. Finally, almost all samples were collected online, which may affect the results compared to in-person experiments. Other researchers might consider the online variables when repeating this study in the future.

### 4.5. Implications

Limitations aside, this study combines the psychological capital theory and COR theory, taking into account ego-resiliency and work-meaning cognition. It enriches the theoretical framework of workplace well-being and promotes our understanding of the effect mechanism of teachers’ psychological capital. Besides the theoretical contributions, the research findings of our research could help teachers improve their workplace well-being with targeted interventions. First of all, we should emphasize strengthening teachers’ psychological capital, such as by holding relevant lectures regularly, attribution training, and group counseling, thereby increasing the cognition of work meaning, and, thus, promoting workplace well-being. In addition, given the role of ego-resiliency, cultivating an optimistic attitude in adversity, thereby increasing the ability of ego-resiliency, would also encourage workplace well-being. In a word, the results of our study provide some feasible advice for promoting teachers’ workplace well-being.

## 5. Conclusions

This study constructed a moderated mediation model to explore the underlying mechanism of how teachers’ psychological capital makes an impact on workplace well-being. The results show that high teachers’ psychological capital could significantly increase the feeling of workplace well-being through work-meaning cognition, and the influence of work-meaning cognition on the relationship between teachers’ psychological capital and workplace well-being is moderated by ego-resiliency.

These findings explore the factors that affect well-being and point to potential ways to enhance teachers’ workplace well-being. Furthermore, these results could promote our comprehension of workplace well-being, which might support the development of effective interventions during daily teaching.

## Figures and Tables

**Table 1:** Demographic characteristics of participants.

Attribute		Frequency	Proportion%
Gender	Male	387	27.90%
	Female	1001	72.10%
Education	Below undergraduate	126	9.08%
	Undergraduate and above	1262	90.92%
Teaching grade	Kindergarten	148	10.84%
	Primary school	768	56.26%
	Middle school	300	21.98%
	High school	149	10.92%
Attribute	All (Frequency)	Mean	SD
Age	1384	39.40	8.84
Working experience (year)	1294	17.93	10.09

**Table 2:** Means, standard deviations, and inter-correlations between the main variables (*N* = 1388).

	*M*	*SD*	1	2	3	4
1. TPC	4.77	0.76	1	*—*	*—*	*—*
2. ER	3.15	0.56	0.60 **	1	*—*	*—*
3. WM	4.36	0.74	0.66 **	0.59 **	1	*—*
4. WWB	4.15	0.77	0.74 **	0.63 **	0.76 **	1

Note: ** *p* < 0.01. Abbreviations: TPC, teachers’ psychological capital; ER, ego-resiliency; WM, work-meaning cognition; WWB, workplace well-being.

**Table 3:** Testing work-meaning cognition as a mediator in the relationship between workplace well-being and teachers’ psychological capital (N = 1388).

Criterion	Predictors	*R*	*R* ^2^	*F*	*β*	Boot LLCI	Boot ULCI	*t*
WM	TPC	0.66	0.43	1061.17 ***	0.65	0.61	0.68	32.58 ***
WWB	TPC	0.82	0.68	1457.91 ***	0.43	0.39	0.47	20.49 ***
	WM				0.51	0.47	0.55	24.10 ***

Note: *** *p* < 0.001. Abbreviations: The test is based on 5000 bootstrap samples to calculate whether the mediating effects are significant at 95%.

**Table 4:** Testing the pathways of the moderated mediation model.

Predictors	Criterion							
	WM(M)				WWB(Y)			
	*β*	*SE*	*p*	95% CI	*β*	*SE*	*p*	95% CI
TPC(X)	0.65	0.02	<0.001	[0.61, 0.68]	0.43	0.02	<0.001	[0.39, 0.47]
WM(M)	*—*	*—*	*—*	*—*	0.51	0.02	<0.001	[0.47, 0.55]
ER(W)	1.00	0.16	<0.001	[0.69, 1.32]	*—*	*—*	*—*	*—*
X × W	−0.12	0.33	<0.001	[−0.19, −0.06]	*—*	*—*	*—*	*—*
	*R*^2^ = 0.706, *F*_(3, 1384)_ = 458.56, *p* < 0.001				*R*^2^ = 0.823, *F*_(2, 1385)_ = 1457.91, *p* < 0.001.			

Note: Analyses conducted using PROCESS model 7, *N* = 1388. Abbreviations: The test is based on 5000 bootstrap samples to calculate whether the mediating effects are significant at 95%.

## Data Availability

The author has specified no data sets for the following reason: The data that have been used are confidential. Due to the sensitive nature of the questions asked in this study, survey respondents were assured raw data would remain confidential and would not be shared.
